# Facilitators and barriers to mental health help-seeking in Indian immigrant youth in Gauteng, South Africa

**DOI:** 10.3389/fsoc.2024.1265353

**Published:** 2024-09-25

**Authors:** Timmy Joji, Curwyn Mapaling

**Affiliations:** ^1^Community Psychosocial Research (COMPRES) Entity, North-West University, Mahikeng, South Africa; ^2^Department of Psychology, University of Johannesburg, Johannesburg, South Africa

**Keywords:** barriers, facilitators, Gauteng, help-seeking, Indian immigrants, mental health, South Africa, youth

## Abstract

**Introduction:**

International literature has documented significant underutilisation of mental health services among Indian immigrants. This study aimed to identify facilitators and barriers to mental health help-seeking among Indian immigrant youth in South Africa by evaluating their personal and lived experiences.

**Methods:**

A qualitative study with a phenomenological design was conducted to understand the lived experiences of Indian immigrant youth regarding mental health help-seeking. Nine participants were recruited through purposive sampling from Gauteng. Data collection was performed through online interviews exploring participants’ lived experiences. Thematic analysis was used to analyse the data.

**Results:**

Five facilitator subthemes were identified: encouragement to seek help for mental health difficulties, social media and mass media influence, university and school environments, availability and awareness of resources, and open conversations about mental health. Four barrier subthemes emerged: individual perspectives on mental health, lack of access to resources, parental factors discouraging help-seeking, and community factors discouraging help-seeking.

**Discussion:**

An improved understanding of these barriers and facilitators may allow other Indian immigrant youth to better manage their help-seeking processes while increasing awareness about similar experiences within the community.

## Introduction

Mental health is an important element of human health and is characterized by an individual being able to cope and be productive. Mental health difficulties on the other hand are a state in which the individual is unable to function well and fulfill the roles and responsibilities that the individual has. Mental health is becoming an increasingly significant problem, with global rates of depression increasing significantly over the last decades.

Mental health difficulties have a widespread impact on individuals from different countries, communities and backgrounds, but some groups are more prone to developing such difficulties. Youth populations, due to increased pressures around academics, independence and finances are one group that is more prone to the development of mental health difficulties (Koutra et al., 2023; Stengård and Appelqvist-Schmidlechner, 2010; United Nations, 2021). Similarly, immigrant groups, due to their cultural backgrounds, are also prone to developing mental health difficulties, with Indian immigrants found to be at particularly high risk (Karasz et al., 2016; Mohammadifirouzeh et al., 2023; Roberts et al., 2016).

A key issue that is relevant to both youth and Indian immigrant populations is the underutilization of mental health services. A lack of help-seeking is prevalent in adult Indian immigrants, and Indian immigrant youth according to the literature (Basri et al., 2021; Chu and Sue, 2011; Kumar and Nevid, 2010; Lee et al., 2009), with both adults and youth often turning to friends or family for support (Lee et al., 2009).

While immigrants were once regarded as grateful members of a community due to their new acceptance and opportunities, research suggests that mental illness may be even more pervasive in these communities. Certain immigrant populations, due to their cultural background and characteristics, are more prone to mental illness than others – with Indian immigrants in particular noted as a high-risk group (Chaudhry and Chen, 2019; Karasz et al., 2016; Roberts et al., 2016). According to the literature, there are significant barriers and facilitators to help-seeking for Indian immigrants, but most studies were noted to focus on barriers (Gulliver et al., 2010). Despite South Africa having a large contingent of international immigrants, no research has been done on the Indian immigrant population and this is particularly concerning when considering the lack of statistics around help-seeking nationally. The experiences of Indian immigrant youth are therefore largely misunderstood, and this may lead many to avoid seeking help. The next section briefly describes the theoretical framework, which underpinned the findings of this study.

### Theoretical framework

Bronfenbrenner’s Ecological Systems theory was selected as the theoretical framework for the study due to its holistic view of the factors influencing an individual’s decisions regarding mental health help-seeking. The theory suggests that the individual’s entire ecological system must be considered when trying to understand their development and has thus been applied in several studies to understanding the ecological influences that may prevent or facilitate mental health help-seeking (Miville and Constantine, 2006; Vogel et al., 2007).

### Research question and aim of the study

This study aimed to explore and describe the potential facilitators and barriers to mental health help-seeking as experienced by Indian immigrant youth. The study focused on answering the following research question: *What are the potential barriers and facilitators for mental health help-seeking of Indian immigrant youth?*

## Method

### Approach and design

The study was conducted with a qualitative approach, which is an approach for exploring and understanding the meaning individuals or groups ascribe to a social or human problem. A phenomenological design was used as the research aimed to understand the lived experiences of Indian immigrant youth regarding barriers and facilitators to mental health help-seeking. The phenomenological qualitative approach to research was the ideal approach to exploring this issue, as phenomenological research is centered on the lived experiences of the participants (Lester, 1999; Neubauer et al., 2019).

### Participants and sampling

The sampling method that was most appropriate for this type of study was determined to be purposive sampling followed by snowball sampling. This was due to the relatively small size of the population of Indian immigrant youth in the Gauteng province that could be recruited using more large-scale sampling methods. Because there were nine participants who joined the study during the first purposive sampling phase, snowball sampling was not conducted to obtain additional participants. Saturation was already reached following the analysis of the interviews of the nine participants recruited through the purposive sampling phase.

The participants for the study were chosen according to the following inclusion criteria. All participants had to be between the ages of 18 and 24 and born of parents with Indian nationality that immigrated to South Africa. The participants must either have been born here in South Africa, or immigrated to South Africa before the age of 10 years old. The participants also must have experienced a mental health difficulty at some point in their life, but did not have to be formally diagnosed, or be in treatment for mental illness before participation. In our study, participants who were not literate and not fluent in English were excluded. This was a necessary criterion due to the nature of the data collection methods employed, which required participants to read and respond in English. We acknowledge this as an exclusion criterion and understand that it may have implications on the diversity and representativeness of our sample.

The potential participants for the study were Indian immigrant youth between the ages of 18 and 24, and as such, it was determined that the best way to go about recruitment would be through community organizations. The South African Malayalee Writers Collective (SAMWRIC) is an organization founded and led by Indian youth who originated from India and grew up in South Africa, and due to their strong presence in the Indian youth community, they were determined to be an appropriate gatekeeper to the community. Following contact between the research assistant (independent person) and the head of the South African Malayalee Writers Collective (SAMWRIC), the founder distributed the recruitment poster to 10 potential participants that were Indian immigrants that fit the age range. Participants had the freedom to decide if they would like to partake, without the need to disclose this to the gatekeeper. Interested participants emailed the research assistant on the email provided in the recruitment poster, and the biographical form was sent to the participants to be completed and checked by the research assistant to confirm participant applicability according to the inclusion and exclusion criteria. The final sample consisted of nine voluntary participants, all of whom were within the 20–24 year age range. Their gender with corresponding pseudonyms are listed below (Table 1).

**Table 1:** Participants’ demographic profile.

Participant	Gender
Roshan	Male
Shreya	Female
Shiv	Male
Mirza	Female
Sita	Female
Divya	Female
Anushka	Female
Kavya	Female
Zaraa	Female

### Data collection

Due to the risks associated with the coronavirus (COVID-19) pandemic, all participants were offered the choice between having an in-person interview, or an online interview over a video call, which would be recorded. All nine participants elected the online interview option.

A biographical form was used to gather the details to confirm that the participant met the inclusion and exclusion criteria for the study. Semi-structured interviews were conducted with the participants. While in-depth interviews are often the norm in phenomenological studies, semi-structured interviews that are flexible, but focused on the topic of barriers and facilitators were regarded as most fitting for the study’s aims. In order to explore and describe the lived experiences of Indian immigrant youth concerning help-seeking, the interview schedule was developed to identify barriers and facilitators that affect help-seeking, as well as the effects of these barriers and facilitators on help-seeking behavior. Field notes were also compiled by the researcher (the first author [TJ]) to maintain objectivity and to encourage the researcher to reflect on personal experiences that may influence their perspectives on the study and the topic at large.

### Data analysis

Before the data could be analyzed, all the recordings were transcribed verbatim, and pseudonyms were assigned to each participant and their interview. A thematic analysis was conducted to identify and evaluate major themes within the answers provided and allowed for the identification of common concerns among the participants (Hsieh and Shannon, 2005). The study focuses on the experiences of Indian immigrant youth in Gauteng and their experiences and does not differentiate between the experiences of participants based on their individual characteristics such as age or gender. Therefore, the thematic analysis approach was regarded as more fitting to the study as opposed to interpretive phenomenological analysis.

In our research, we adapted Braun and Clarke’s (2006) thematic analysis steps to suit the specific needs and context of our study. Initially, we familiarized ourselves with the data by listening to audio recordings and transcribing the interviews, akin to Braun and Clarke’s first step of data familiarization. During this phase, we took detailed notes to identify broad themes that stood out, which reflects the second step of generating initial codes.

In the subsequent stage, we determined initial codes by evaluating the transcribed data. This process involved categorizing the data into descriptive segments, a personal adaptation of Braun and Clarke’s coding step, where we focused on the nuances specific to our study’s context.

Next, we identified themes based on these codes, allowing for a more interpretive and broader perspective, aligning with Braun and Clarke’s fourth step. This was where our study’s unique focus became evident, as the themes we identified were tailored to the specific experiences and narratives of our participants.

Further, we refined these themes by evaluating the codes under each category for overlaps or multifaceted elements, which is a personalized take on Braun and Clarke’s fifth step of reviewing themes. This involved a detailed examination of how different codes interconnect.

Finally, in the last step of defining and naming themes, we provided a comprehensive description of each theme’s story and its contribution to the study. This step formed the backbone of our article, with direct quotations from the collected data included to reinforce the themes’ meaning and significance, as advised by Braun and Clarke.

By adapting Braun and Clarke’s methodology in this manner, we ensured that the thematic analysis was not only rigorous but also deeply reflective of our study’s unique context and objectives.

### Trustworthiness

The study’s credibility was enhanced by the interviews, which allowed for prolonged engagement to understand participant experiences, which were transcribed verbatim. The researcher conducted the coding and analysis himself, while also using an experienced and objective co-coder [a Health Professions Council of South Africa (HPCSA) registered research psychologist]. The transferability of the study was enhanced by the researcher’s focus on the overarching goal of understanding the barriers and facilitators related to mental health help-seeking, allowing the findings to be potentially transferable to groups other than the one being studied. The study had a clear process, which was followed meticulously and stems from the aims and questions that needed to be answered to achieve the goals of the research, thus improving dependability. Lastly, the confirmability of the study was enhanced through a clear discussion of the conclusions and inferences that were reached through the interpretation of the findings.

### Ethical considerations

The ethical approval for this study was obtained through the Health Research Ethics Committee (HREC) of the North-West University (ethics approval number: NWU-00022-22-A1). Goodwill permission to conduct the study was obtained by contacting the leader of the SAMWRIC. Interested participants emailed the research assistant anonymously and arranged video calls to discuss the study and informed consent, agreeing that they would be allowed to keep their video off to protect their identities and would only need to turn on their video to display their signing of the informed consent form. Verbal consent was also once again obtained at the start of the interviews, which were conducted via the Zoom platform.

The study itself had a certain level of risk as the population that was being interviewed is viewed as a vulnerable population, and safeguards were put in place to protect them further. To manage this risk, a trained lay counselor from the South African Depression and Anxiety Group (SADAG) was available to provide support, and an HPCSA registered clinical psychologist was also arranged and was available after each interview for a video call to provide more in-depth assistance in the form of debriefing. Each participant was also provided with the details for the SADAG’s 24-h suicide helpline, as well as SADAG’s crisis intervention line for the participant to be able to access telephonic counseling and crisis intervention, if needed.

## Findings and discussion

On the basis of the data, a thematic analysis was conducted, leading to three themes being discovered regarding the experiences of Indian immigrant youth concerning help-seeking (see Table 2). Verbatim quotations are provided to support the themes and subthemes that were discovered.

**Table 2:** Themes and subthemes.

Theme 1: Factors contributing to the mental health difficulties experienced by Indian immigrant youth
Subtheme 1.1: Academic stress
Subtheme 1.2: Relationship difficulties
Subtheme 1.3: Social stressors
Subtheme 1.4: Adjustment difficulties
Theme 2: Factors that serve as facilitators of help-seeking in Indian immigrant youth
Subtheme 2.1: Sources of help
Subtheme 2.2: Encouragement to seek help for mental health difficulties
Subtheme 2.3: Social media and the mass media
Subtheme 2.4: University and school environments
Subtheme 2.5: Availability and awareness of resources
Subtheme 2.6: Open conversations about mental health
Theme 3: Factors that serve as barriers that prevent or discourage help-seeking in Indian immigrant youth
Subtheme 3.1: The individual’s perspectives on mental health
Subtheme 3.2: A lack of access to resources
Subtheme 3.3: Parental factors that discourage help-seeking
Subtheme 3.4: Community factors that discourage help-seeking

Three main themes were identified from the data, namely (i) factors contributing to the mental health difficulties experienced, (ii) factors serving as facilitators that encourage help-seeking, and (iii) factors that serve as barriers that discourage help-seeking. There were also some significant consistencies in the experiences of the participants, with most participants reporting that they knew that they were going through mental health difficulties but were not able to self-diagnose or evaluate the severity of their difficulties accurately – something that often led to several participants deciding that their difficulties are not serious enough to warrant help. Many participants reported that they first spoke to a parent about their desire to seek help, and often did not receive support for their decision. The responses from the participants also made it clear that for Indian immigrants, and particularly their parents, Indian culture plays a significant role in guiding decisions about career paths and mental health help-seeking. As a result, many first engaged in help-seeking after leaving home, reporting that the university environment allowed them more personal agency to seek help, even when doing so privately. Participants largely felt that help-seeking would be beneficial for someone going through mental health difficulties, but the responses also indicated that the barriers preventing help-seeking seemingly outweigh the facilitators that encourage help-seeking. The themes and subthemes were identified based on the research question, have been listed in Table 2, and are discussed in detail thereafter.

## Theme 1: factors contributing to the mental health difficulties experienced by Indian immigrant youth

The first theme involved the factors contributing to the mental health difficulties experienced by the participants.

### Subtheme 1: academic stress

Many participants reported experiencing anxiety relating to their academics, and several participants reported that this academic stress was further related to prescriptions about which careers are good or bad. Work anxiety was often compounded by other stressors and COVID-19-related changes to the academic environment. This was indicated by Mirza, who said, “*So, for most of my first year, I was thinking that I might drop out. It was mostly the fact that I make the wrong choice. Should I drop out? Will I still be able to carry on with this degree*”?

### Subtheme 2: relationship difficulties

Participants indicated that their mental health was impacted by their relationships and the pressure of balancing relationships with other responsibilities. Some participants experienced difficulties in their relationship with their family or parents - often feeling unhappy with the lack of support they receive, or the prescriptive nature of their parents when it comes to career decisions, friendships, and appearance. One participant, Shreya shared, “*Then in terms of other pressures, like relationships: Whether it’s like friend relationships, romantic relationships, again even family relationships [can be a source of stress]*”.

### Subtheme 3: social stressors

Indian immigrant youth reflected on how social stressors, such as trying to fit in, making new friends, and coping with bullying contributed to their mental health difficulties. Roshan shared the following:

*If it’s not the studies, then it's going to definitely be about making friends and getting into the university environment. Let's say people bully you and things – you can’t really tell your parents about this. Because as ones from the first generation, they will just be like, just get over it. You know?*

### Subtheme 4: adjustment difficulties

Participants reported that adjusting to moving away from home and into the university environment was a significant contributor to their mental health difficulties as they often found it difficult to be responsible for themselves for the first time while juggling their academic demands, social lives, and health. Participants also reflected on how a sheltered upbringing can also lead to individuals rebelling or exploring for the first time at university. Roshan reported as follows:

*It’s because, as a young adult studying in the university, we get a lot of pressure from the university with work and trying to balance our social lives, our going out, our playing sports and exercising*.

## Theme 2: factors that serve as facilitators of help-seeking in Indian immigrant youth

The second theme that was identified was the factors reported by the participants to encourage or facilitate help-seeking and included the prominent sources of help that were reported by them.

### Subtheme 1: sources of help

Participants reported that cousins, and in some cases, parents were a source of support for them, though participants reported that mothers were considered closer and more understanding. Almost half the participants reported that they turned to their friends for support, with some reporting that they decided to not see a psychologist when they found that their support from friends was sufficient to help them to manage their mental health difficulties. Mirza shared*, “I eventually just spoke to friends and then that helped and that kind of took off the psychologist thing. They’re also going through it as well so they can also relate to it”*.

### Subtheme 2: encouragement to seek help for mental health difficulties

Almost half the participants reported that having the support and encouragement of siblings and friends and parents to seek help for their mental health difficulties provided a push to seek help. Anushka reported, *“Whenever I was with her [my sister], it gave me so much more openness to any facilities that would help my mental health”*.

### Subtheme 3: social and mass media

Some participants reflected on the role that the media and social media play in displaying help-seeking as something that is necessary and helps to encourage peer support. Divya shared, *“Also, these days there are a lot more movies surrounding mental health, which I think is very cool.”* Similarly, Shreya said, “*So, I think if we make Facebook groups to be like - listen, I have a problem and I would really appreciate somebody to listen to my problem and maybe give their perspective about this issue”*.

### Subtheme 4: university and school environments

The university environment was widely regarded as a place where almost half the participants were first exposed to mental health knowledge and promoted autonomy and free-thinking, as indicated by Divya, who stated the following:

*At the same time, I think a lot of times when you leave home to go to university and you're in your own space, you're exposed to a lot more. It does open up a lot of people and it does encourage them to get the help that they need*.

Participants also felt that the school environment could play a significant role in promoting mental health knowledge and normalizing help-seeking from a young age. Shiv shared, *“So to try and create awareness of it in a primary school level. I think, that would make it easier for them to talk about mental health difficulties”*.

### Subtheme 5: availability and awareness of resources

Most of the participants that did see a psychologist reflected on how the affordability and convenience of seeing the campus psychologist encouraged them to seek help. The fact that parents would not be aware of their help-seeking compared with if they saw a private psychologist using their medical aid, also encouraged them to seek help. This perception was expressed by Sita:

*“Yeah, it was convenient. And the fact that I didn’t have a car at the time. So even if I went to private counselling, it will go through medical aid. And my parents would know because I did bring it up”*.

### Subtheme 6: open conversations about mental health

Participants reflected on community support around mental health help-seeking, and how this would encourage youth to seek help and create an environment for shared resources and knowledge. Shreya stated, *“I think having that community, it’s a nice step in helping somebody get sent to the help that they actually need. If there’s a community that’s actually there to help them and to guide them in the right direction”.*

Participants, however, did feel that the most significant community factor that would promote help-seeking would be to see other community members also seeking help and normalizing the process. This perception was expressed by Mirza:

I feel I'm very scared of judgment, and if more people are open to it then I don't think anyone's going to judge you for it. Because you're not the only one that's doing it. So, it won't make you seem like you're weak. So if everyone does it, then that’s something that I won’t be judged about.

Participants felt that youth and community conversations regarding mental health would further normalize help-seeking. Zaraa shared, *“And I think relating your own experience, even if it’s I do not know if it’s the right way, but like, I think that definitely encourages someone else. Because then they are like I’m not the only one”*.

Participants believed that a youth community that promotes mental health and help-seeking for mental health difficulties would also be taken more seriously in the greater community and by parents, and potentially begin a greater communal discussion about mental health, as opposed to leaving it as a taboo topic. Anushka shared the following:

*I’m trying to think how you would like force all the aunties and uncles to just like, sit and talk about it. But, maybe even if we can, with the adults, have a consensus of being together and being united to fight that mental health help should be a priority. And to then take that back to our parents with that kind of unity as the youth*.

## Theme 3: factors that serve as barriers that prevent or discourage help-seeking in Indian immigrant youth

The third theme that was identified relates to the factors that participants felt were barriers that discouraged or deterred them from seeking help.

### Subtheme 1: the individual’s perspectives on mental health

Participants often wanted to manage their mental health difficulties themselves – partly because they believed that this would help them grow, or because they would find it easier to take their own advice. Additionally, most of the participants were noted to minimize the severity of their difficulties and were also noted to do this often while comparing their own situations with those of others who they deemed to have bigger problems than their own. Mirza made the following comment:

Yeah. Every now and then whenever it was getting worse I’d think, maybe I should. But then I just left it and asked myself is it really necessary? Their problems are so much bigger than mine, so mine is so much smaller and there's no point in getting that help.

While many participants minimized the severity of their difficulties, they reflected on still wanting more support from their families. Roshan stated, “*You want to fight your own battles and stuff, but you actually really want more support from them.”*

Many participants also acknowledged and reflected on how parental and old perspectives are often passed down and internalized, particularly relating to mental health. Shreya stated, “*And for that reason, they sort of have passed that on to their children where they do not see the need to address mental health.”*

### Subtheme 2: a lack of access to resources

A few participants noted that not everyone knows about the available resources and thus they do not use them. Other participants reported that while they knew about the available resources, they were unable to use them as they could not pay for a psychologist themselves after their parents disapproved. Divya stated as follows:

Because, our parents do pay our fees they get all the documents and all of that stuff. But then I think that also stops kids from getting the help they need from the university because they're so scared. Oh, my God, what if my parents find out that I am getting help?

### Subtheme 3: parental factors that discourage help-seeking

Most participants reported that their parents have strong views about mental health and help-seeking and felt that community members will also judge individuals that seek help. Participants reported that depression is viewed as insignificant, and something that can be overcome personally. Kavya reported, “*Depression is just being really sad, in their eyes, and you can get over being sad. Because if they can do it, why cannot we?*” Mental health help-seeking, however, was often associated with the idea of severe mental health problems. Divya stated, *“Okay, if you are seeing a psychologist, they think that only someone that’s crazy needs a psychologist”*.

Participants reported that their parents were concerned about the community becoming aware of their mental health difficulties for fear that their child would be judged as crazy, and that they may be viewed as inadequate parents and shamed. Sita reported, “*I think they have this fear that if society finds out that – oh my God, my child is fighting depression, or my child is fighting anxiety, it somehow reflects badly on them*”.

Fear of the community finding out about their child’s mental health help-seeking led parents to minimize the severity of the participant’s experience or by suggesting that help is received inside the family from themselves or siblings rather than externally, potentially due to them feeling inadequate or as though they have failed. Sita remarked as follows:

*And something I also noticed is that they always avoid the fact that you need to seek help elsewhere. Maybe on some level, it makes them feel like they haven't done a good job. Like, if my parents need to send me to therapy, then it means they failed as a parent*.

Parents’ fear of community stigma was also related to fear that their child would be ostracized in the community or be viewed as a poor choice as a bride or groom. Sita remarked as follows:

*And there was a phase in my life where I needed therapy, and then I did mention it to my parents. And they were very much like, you know, don't push yourself to the point where you need therapy, so you don't need that kind of thing. Yeah, that it’s gonna affect your future, it's gonna affect how people see you*.

The emphasis on image may be understandable considering how participants reflected on how their parents immigrated and built successful lives and were able to do so without seeking help for their mental distress. Roshan commented as follows:

*Yes, there is because for us, there's a set path that our parents said we should follow. They migrated from India, went to another country to get a better job. They got a wife or husband and then they settled down. They want their child to do much better compared with what they have done*.

This resulted in participants feeling as though they were required to also be successful without complaining and requiring mental health assistance, which their parents never had. Zaraa said, *“My mom said; in our days, like there was nothing – like you cannot even tell your parents these things. She was like, but I’m here, you can talk to me”*.

Many participants, therefore, avoided disclosing problems to their parents for fear they would disagree about them seeking help. Sita shared, “*But I was so scared that they’d disagree and I’d just suffer in silence–- that I just decided to do it on my own”*. Participants rather chose to seek help though university structures. Kavya said, *“But I always knew once I got out, I would be able to. When things got very hard. I always feel like it will get better. Once I’m in* var*sity and I have more freedom”.*

### Subtheme 4: community factors that discourage help-seeking

Participants reported feeling as though the Indian immigrant community did not have a good understanding of mental health, and help-seeking was not normalized or understood. Sita made the following statement:

*And so they just suffer in silence, or they isolate and that causes a bigger problem. And then it probably pushes them to the point of even suicide. And then the parents wonder, like, what did I do wrong? And then it's too late. So I think one of the main barriers is the moment someone says mental something, they just shut down*.

Participants also reported that parents and the community placed a very high value on academic success throughout a young person’s life. Roshan said, “*But in the Indian society we mostly want doctors or engineers. And then we want them to marry another doctor or engineer just to help society, and to get money.”* This emphasis on achievement was reported to start with high standards of performance in school and continue into university, with a focus on an academic comparison between children in the same age group. Shreya shared the following:

*My parents will be comparing my sister with everyone in her age group, or me when I was in high school and everyone in my age group. And like, ‘did you hear what this girl did, did you hear what that girl did, did you hear she got she graduated with cum laude?’*

Participants also reported high standards for strength and resilience, or risking being viewed as weak by the community – something that held significant value for both participants and their families. Kavya said, “*And I was scared of telling them because they might. Yeah, like, try get me to not go. Also feel judged like: Oh, are you that weak that you need to talk to someone?”*

Some participants were concerned that their mental health difficulties will be openly discussed with the extended family or the community, and they would be viewed as flawed. Shreya said, *“And then the community knows, and all of a sudden, I’m labelled as that problem child who’s looking for counselling. That definitely is a barrier.”* Additionally, several participants reported that they believed that their image or reputation would be affected by the community knowing that they are experiencing mental health difficulties and seeking help. Anushka shared the following:

*So you don't want that to go around. You don't want this image of someone who's damaged and depressed or anxious or whatever, to be your image and to like, maintain that. It sounds silly but you don’t want the aunties to be like: Oh, my gosh, Anushka was admitted in the hospital*.

Through both the background literature as well as the findings of the study, it is clear that culture plays a significant role in help-seeking decisions. This is in line with the current perspectives on the impact of culture nationally, as there are significant culturally rooted differences in how mental health and mental illness are conceptualized, judged, and treated. As a result, the evaluation of the findings required a systemic perspective that acknowledges the impact of culture on different spheres of life, such as Bronfenbrenner’s Ecological Systems theory.

The identified subtheme above and the other subthemes and themes show that elements of the microsystem, namely parents, friends and peers, the university environment and health services have a prominent impact on help-seeking decisions, with parents perhaps playing the most prominent role. Participants discussed how parents often lacked knowledge about mental health and often viewed mental illness as something that should be hidden. Most participants agreed that there was pressure associated with trying to live up to their parents’ achievements in coming to a new country and being successful and resilient, leaving many participants feeling that they had to display the same resilience and be equally successful. Participants, therefore, felt that their local health systems or private psychologists were not an option as they feared that their parents would find out, leading many to seek help in secret to avoid their parents finding out. Friends, cousins, and peer groups played a significant role as a source of help in that participants often turned to friends for support and found this to be helpful due to their ability to relate to similar experiences. The university environment contributed to some participants’ mental health difficulties due to academic stress but was also the first place where many participants became aware of mental health and found help-seeking to be something normal for people to do.

There were also notable interactions between the different elements mentioned above, which fall into the sphere of the mesosystem. These were centered around the parents, and their interaction with either their friends or the university. Participants felt that their friends seeking help may normalize the process for their parents and make it easier for them to seek help. The interactions between parents and the university were also significant, as participants felt that because their parents pay their university fees, they may be privy to information about their child’s help-seeking when using university support services.

Elements of the exosystem, such as the role of extended family and the media, were also present in the barriers and facilitators identified, as participants were concerned that friends of the family might become aware of their help-seeking and disapprove. Participants reflected on how there are more positive depictions of help-seeking in the media nowadays, and how social media could connect and unify Indian immigrant youth who want to converse about mental health and help-seeking and provide support to each other without judgment.

The macrosystem, made up of the elements that are common to a specific culture, plays a significant role in the help-seeking decisions as participants’ parents and the community at large are still very connected to traditional Indian culture. Indian parents and the community were reported to put a huge emphasis on achievement at school, university, or their career, which connected to finding good marriage proposals. Participants also reflected on the pressure to always be strong and resilient, characteristics that were valued by the community of immigrants that had to move to a new country and become successful. Many participants were noted to minimize their mental health difficulties by suggesting that they should be strong enough to overcome it, or that the issue is not significant compared with the difficulties others face, leading to many deciding not to seek help.

The ecological systems theory therefore provided structure to the various barriers and facilitators that exist in the various participants’ experiences. The microsystem and its elements were certainly the most prominent direct influence, but because the macrosystem encompasses the microsystem, the behavior and decisions of various individuals in the microsystem were significantly influenced by the macrosystem. Standards of resilience, strength, stability, and success in the Indian immigrant community had led to many participants feeling overwhelmed by the responsibility to appear as though they are thriving, while also denying the need for help for fear that they will not appear independent or resilient if they are assisted.

Elements in the microsystem and exosystem also contributed as facilitating factors, with friends, cousins and peers providing support and normalizing help-seeking. Lastly, social media was viewed as a possible tool moving forward with which to connect Indian immigrant youth and provide a space for advice and support, while also promoting mental health and help-seeking to the Indian immigrant community at large.

Through the thematic analysis, four categories of barriers and five categories of facilitating factors were identified. The findings of the study are largely in line with the existing literature, with barriers such as a lack of knowledge about mental health, norms associated with family support, the significance of family privacy, and stigma, all featuring prominently in the themes identified in the study. However, there was a lack of existing studies and background literature on factors that facilitate help-seeking in Indian immigrants.

The facilitators to help-seeking identified through the thematic analysis were, therefore, not anticipated and served to highlight the significance of a support system that encourages help-seeking; university environments and other environments that encourage free thinking and make help-seeking resources available. Moreover, the data analysis findings revealed the importance of open conversations normalizing mental health difficulties and the positive role that the media and social media can play in this process.

### Implications for the South African context

The participants in the study discussed the facilitators that encourage help-seeking and barriers that prevent help-seeking in the Indian immigrant youth population. Immigrant populations, and specifically Indian immigrant populations, have displayed low rates of help-seeking for their mental health difficulties, according to the existing literature. This pattern was also observed in the participants that took part in the study, with many either minimizing their difficulties or attempting to overcome their difficulties on their own and choosing not to seek help despite their largely positive perceptions about help-seeking and therapy. Through the study, it has also become clear that the influence of parental and community perspectives on mental health has had a significant impact on the participants’ decisions to seek help. While there were also various facilitators identified, the impact of the four categories of barriers outweighs them; leading to many participants displaying a disinclination to seek help.

This suggests that there may be many Indian immigrants across the country who are experiencing similar difficulties in seeking help for their mental health difficulties and suggests that there is a strong need for change. As suggested by one participant, such change would have to take the form of open conversation about mental health and help-seeking; something, which many participants believe does not happen in the home or the community. Several participants also reflected on the importance of young people taking responsibility for collaborating, communicating and providing support through social media platforms, in the hope of encouraging Indian immigrant youth to seek help, which in turn normalizes the process for others as well.

Considering the prominent immigrant population in South Africa, along with the known impact of culture on mental health perspectives, it is also clear that interventions to improve help-seeking rates must consider cultural background. It was noted in both the literature as well as in the participant responses that a culturally sensitive approach to understanding mental illness improves the individual’s ability to speak openly and feel understood. National policies around mental health awareness should therefore tailor intervention programs, and awareness programs to different cultural perspectives as opposed to taking a one size fits all approach for all backgrounds and cultural contexts. Such policies should be guided by future research, which should further explore the complex relationship between stigma, social dynamics, and the family system that influences decisions to seek help – something that is lacking in the extant literature. Studies that also provide a more balanced sample with a greater representation of male participants may also be of value, as the small number of male participants in the current study may be reflective of gender norms around help-seeking, and may provide new insights into the perspective of male Indian immigrant youth.

It is hoped that an improved understanding of the barriers and facilitators will also allow other Indian immigrant youth to manage their own help-seeking process and be more prepared for possible obstacles, while also being aware of how many other youths also have similar experiences regarding their mental health and help-seeking. Lastly, it is also hoped that parents become more aware of the complexities and challenges of their children’s experiences and encourage and normalize help-seeking. It is hoped that, in turn, this will lead to a community that is more accepting of mental health difficulties and encourages the youth to seek help.

## Limitations

The limitations of the study included the demographics selected and the location. Indian immigrant youth in smaller cities or rural areas may have a different experience, with specific barriers and facilitators having a more pronounced effect on their lives. The participant sample in this study exhibited a notable gender imbalance, predominantly comprising female participants, with only two male participants included. This disproportionate representation may have introduced a bias, potentially leading the findings to more closely reflect the experiences and perspectives of female participants, thereby limiting the generalisability of the results across different genders. Additionally, the barriers and facilitators identified in the study may not be applicable or consistent with the experience of non-immigrant youth, or even immigrant youth from other countries that bring their own cultural perspectives, which may be vastly different to those of Indian immigrants.

## Conclusion

This study aimed to explore and describe the potential barriers and facilitators for mental health help-seeking experienced by Indian immigrant youth. The findings of the study suggest that several barriers and facilitators influence an Indian immigrant youth’s decision to seek help. The findings also suggest that the barriers currently outweigh the facilitators, and much Indian immigrant youth may therefore avoid help-seeking due to issues such as a fear of judgment from parents and the community or a fear of appearing weak, crazy or as though they are not resilient. Many participants were also noted to minimize the severity of their problems by making comparisons to others who are in worse states of mental health or making comparisons to the difficulties their parents faced as immigrants and their resilience. Most participants felt that mental health conversations are not common in the Indian immigrant community, thus further stigmatizing the process of help-seeking. Participants agreed that change was necessary, but also felt that this may need to start in the youth. Youth groups that could provide support, and advice, and open up the conversation on topics of mental health and help-seeking were regarded as a possible first step toward normalizing mental health. Additionally, participants felt that a group of youth with the same message around normalizing mental health and help-seeking might have a stronger impact on the perspectives of the Indian immigrant community at large, as opposed to isolated conversations at home with parents. A shift in perspective from the adult population in the Indian immigrant community to open up to conversations about mental health would better allow the community to understand the complexities and challenges of the experiences of the youth and encourage them to not only find but to access the necessary help.

## Data Availability

The raw data supporting the conclusions of this article will be made available by the authors, without undue reservation.
